# Signals of forest degradation in the demography of common Asian amphibians

**DOI:** 10.7717/peerj.4220

**Published:** 2018-01-31

**Authors:** Nancy E. Karraker, Samantha Fischer, Anchalee Aowphol, Jennifer Sheridan, Sinlan Poo

**Affiliations:** 1 Department of Natural Resources Science, University of Rhode Island, Kingston, RI, United States of America; 2 Natural Resources Institute, University of Manitoba, Winnipeg, Canada; 3 Department of Zoology, Kasetsart University, Bangkok, Thailand; 4 Department of Environmental Studies, Yale-NUS College, Singapore; 5 Department of Conservation and Research, Memphis Zoo, Memphis, TN, United States of America

**Keywords:** Amphibians, Body condition, Demography, Predation risk, Survival, Hong Kong, Thailand, *Duttaphrynus*, *Microhyla*, *Polypedates*

## Abstract

**Background:**

Lowland areas in tropical East and Southeast Asia have a long history of conversion from forestland to agricultural land, with many remaining forests being chronically degraded by wood cutting, livestock grazing, and burning. Wetland-breeding amphibians that have evolved in lowland forests in the region have adjusted to changes in habitat composition caused by humans’ activities, and populations continue to persist. However, we have little understanding of the impacts of forest disturbance on these species beyond assessments of abundance and distribution, and species considered to be common and widespread have been largely neglected.

**Methods:**

We examined body condition and sex ratios of toads (*Duttaphrynus melanostictus*), predation risk in treefrogs (2 *Polypedates* spp.), and growth and survival of leaf litter frogs (2 *Microhyla* spp.) in agricultural land, degraded forest, and intact forest in two study areas, Thailand and Hong Kong.

**Results:**

Toad populations exhibited higher body condition and female-biased sex ratios in intact forest. Predation of treefrog embryos by flies was lower in intact and degraded forests than in agricultural land. Embryonic survival and larval growth and survival in leaf litter frogs were lower in intact forests than in agricultural land. Results for each study were similar between study areas.

**Discussion:**

For three of five of these common amphibian species, we documented signals of forest loss and disturbance in their populations. Although these species occur in disturbed habitats, loss of forest cover continues to degrade aspects of their population demography. We urge conservation biologists to consider that populations of species appearing to be common, widespread, and tolerant of human disturbance may be eroding over time.

## Introduction

Conversion of forest to agricultural land has been the greatest contributor to habitat loss in terrestrial ecosystems (Pereira et al., 2010) and has been the main driver of land conversion in Southeast Asian forests in recent decades (Achard et al., 2002). Populations of a broad array of species have been negatively affected by forest loss and degradation in Southeast Asia (Laurance et al., 2012). For example, over 50% of bird species have been extirpated in Singapore, which has lost 95% of its lowland rainforest since the late-1800s (Castelletta, Sodhi & Subaraj, 2000). In Thailand, near-complete extirpation of small mammals occurred within 25 years of fragmentation of forest into habitat islands (Gibson et al., 2013). Studies of species’ responses to habitat loss and degradation have most often assessed changes in species diversity or abundance, yet more subtle effects associated with habitat changes may also act as stressors on populations (Cushman, 2006). With chronic conversion of forest to agricultural land over time and long histories of disturbance to remaining forests, particularly in lowland areas of tropical East and Southeast Asia, impacted populations may exhibit changes in important biological and ecological attributes that may compromise viability over time.

Many amphibian species inhabiting lowland areas in tropical East and Southeast Asia are considered to be common and widespread, despite the fact that population studies are scant or non-existent and that phylogenetic relationships are not well-resolved for most taxa. The notion that rarity is common and commonness is rare (sensu Gaston, 2010), meaning that a larger proportion of species are rare than are common, applies as well to lowland species in this region of Asia, as it does to species in North America and Europe. Common species in many ecosystems contribute strongly to community structure (Gaston & Fuller, 2008), biomass (Burton & Likens, 1975), nutrient cycling (Vanni et al., 2002), and energy transfer between trophic levels (Gibbons et al., 2006), often serving important roles as both predators and prey. Yet the roles of amphibian species considered to be abundant and broadly distributed in this region of Asia are understudied and assumptions are made that these species are highly tolerant of disturbance and will continue to thrive. Rightly, conservation funding and action have been directed at species considered to be at risk of decline or extinction (Lindenmayer et al., 2011), but the near-complete disregard for the viability of populations of ‘common’ species is unwarranted given their importance to ecosystems.

Forest loss and degradation have been shown to impact the demography of amphibians in other regions. Research in North America suggests that forest disturbance affects body condition (Karraker & Welsh Jr, 2006) and survival (Skelly & Freidenburg, 2000) of forest-associated amphibians. The paucity of studies on mechanisms leading to changes in abundance and diversity signal a need for further research to delineate the means by which forest disturbance impacts amphibian populations, particularly outside of North America. Our aim was to answer the following research questions: (1) How do forest loss and degradation affect demographic attributes of populations of common frogs? (2) Are those effects consistent among different locations? We answered these questions by conducting three identical studies in two different study areas, northeastern Thailand and Hong Kong, China. In each study area, we compared population attributes of three frog taxa in intact forest, degraded forest, and agricultural land. Specifically, among the three habitat types, we compared body condition and sex ratio in populations of the Asian common toad (*Duttaphrynus melanostictus*), embryonic survival and larval growth and survival of the ornate pygmy frog (*Microhyla fissipes*) in Hong Kong and its congener the dark-sided chorus frog (*M. heymonsi*) in Thailand, and predation risk by fly larvae on embryos of the brown tree frog (*Polypedates megacephalus*) in Hong Kong and its congener the common tree frog (*P. leucomystax*) in Thailand. Little information is available on their densities, and populations sizes have not been estimated for any of the species. In Taiwan, reported mean densities were 0.07 frogs/100 m^2^ for *M. heymonsi*, 0.17 frogs/100 m^2^ for *P. megacephalus*, and 3.27/100 m^2^ for *Duttaphrynus melanostictus* (Huang & Hou, 2004). In Thailand, mean densities of all forest floor frogs, presumably including *M. heymonsi* and *D. melanostictus* ranged from 0.12–0.27/100 m^2^ between habitat types (Inger, 1980). However, researchers have not previously compared population attributes among habitats with different levels of degradation. We focused on this set of species because they are considered to be common and widespread and have been regarded as ‘human commensals’ (Heinsohn, 2003) because of their presence in disturbed habitats. However, their occurrence in disturbed habitats may belie impacts to other population-level attributes that warrant investigation.

## Materials & Methods

### Study area

In Thailand, we conducted research at Sakaerat Environmental Research Station in Nakhon Ratchasima Province from July–October 2010 and August–September 2011. In Hong Kong, we conducted our study in the New Territories and Lamma Island from April–September 2011. In Thailand and Hong Kong, our research occurred in intact secondary forest, degraded secondary forest, and agricultural land. In Thailand, intact forest was structurally complex, mature, dry evergreen secondary forest with legacy trees up to 400 years old. This forest type is dominated by plants in the family Dipterocarpaceae including *Hopea* spp. and *Dipterocarpus* spp., and generally exhibits a dense shrub and liana layer and a closed canopy layer. Degraded forest was dry deciduous secondary forest, dominated by Dipterocarpaceae including *Shorea* spp. and *Dipterocarpus* spp., with a single canopy layer and grass-dominated understory that is burned annually (Inger & Colwell, 1977; Tanaka et al., 2008). Agricultural land consisted of wet agricultural areas in which rice (*Oryza sativa*) and water lotus (*Nelumbo nucifera*) were grown. In Hong Kong, intact forest was structurally complex, mature, deciduous-evergreen secondary forest, with most important canopy trees being *Persea* spp (Lauraceae) and *Schefflera octophylla* (Araliaceae) (Kwok & Corlett, 2000). This area had been completely deforested by the 17th century, but re-growth began after World War II (Zhuang & Corlett, 1997). Degraded forest was mixed deciduous-evergreen secondary forest with a single canopy layer and an herbaceous understory that is regularly disturbed by human-caused fire. Agricultural land was an area of rice paddies and other wet agricultural crops, including water cress (*Nasturtium officinale*) and water lotus (*Nelumbo nucifera*). To our knowledge, frogs are not collected by humans in any of the study areas.

### Toad body condition and sex ratio

Surveys for toads were conducted after the breeding season from August–October 2010 (start of the wet season) in Thailand, where the species is known to breed from December–March (Heyer, 1973) and in Hong Kong from July–September 2011 (wet season), where the species breeds February–May (Lau, 1998). This timing was intended to ensure that both sexes occurred in all habitats and females were not gravid. We conducted surveys in each habitat type beginning 30 min after dark. Distances of approximately 2 km were surveyed along different trails each night, and we alternated habitat types on subsequent surveys. Seven transect searches were conducted within each habitat type (intact forest, degraded forest, and agriculture) in Hong Kong and six transect searches were completed in each habitat type in Thailand. We captured all adult toads observed during transect searches and incidental toads encountered during project-related activities in each habitat type. Toads were measured and weighed, and sex was determined. Male *D. melanostictus* reach sexual maturity by 50 mm in size (Huang, Lin & Yu, 1997; Ngo & Ngo, 2013), and we used this as a lower size limit for making a sex determination. Sex in this species can be determined by relative size of forelimbs, which are notably larger in males even outside of the breeding season, and by presence of nuptial pads, which often remain apparent beyond the breeding season. Individuals with ambiguous characters were recorded as of unknown sex. The same two researchers in Hong Kong and the same two researchers in Thailand, all of whom had previous experience determining sex of *D. melanostictus* in the field, made all sex determinations. Toads were marked by clipping the distal 1/5 of a single toe. We calculated a body condition index for individuals in each habitat type using the residuals from a linear regression of body mass against total length (Schulte-Hostedde et al., 2005); a positive mean residual indicates higher body condition and negative mean residual indicates lower body condition (Reading, 2007). Body condition indices for toads were compared using a general linear model with habitat type and sex as factors. Length and mass data were log_10_-transformed to meet model assumptions. We determined sex ratios and compared them with an assumed even sex ratio using chi-square for each habitat type.

In Hong Kong and in Thailand, we established 15, 25-m^2^ plots within each habitat type at randomly selected directions and distances up to 100 m from surveyed trails. We measured percent overstory canopy cover, tree diameter at 1.1 m above the ground for trees ≥5 cm, and heights of three dominant trees. We compared characteristics among habitat types using *t*-tests.

### Growth and survival of leaf litter frogs

We studied growth and survival of leaf litter frogs from August–September 2010 (start of wet season within the breeding period for the species) in Thailand, where *M. heymonsi* breed from February–October (Heyer, 1973), and from April–June 2011 (wet season) in Hong Kong where *M. fissipes* breed from March–August (Lau, 1998). In Thailand and Hong Kong, we established arrays of 18, 30-liter containers, grouped as three sets of six, in each habitat type. As such, we had three replicates each within intact forest, degraded forest, and agriculture in each study area. Each set was located 100 m apart. To each container, we added 24 liters of pond water, filtered through 0.5 mm mesh, and 10 g of dry leaf litter. Within each container, a 2-liter plastic container with 1 liter of water was floated on the surface to hold developing embryos. Larger containers were covered with plastic mesh (1 cm^2^ openings) to exclude predators. We collected six egg masses of *Microhyla* spp. from nearby ponds. Each egg mass was divided into thirds and one-third was placed in the small plastic container in each larger container. After hatching, in two to three days, we transferred 25 hatchlings from the smaller to the larger container. We counted all live tadpoles, undeveloped eggs, and dead embryos, and calculated proportion of eggs surviving to hatching. Larvae of *M. fissipes* generally metamorphose within 25 and 30 days (Ma, 2012) and it was suspected that duration of larval stage was similar in *M. heymonsi*, so we terminated the studies at 21 days. We counted the number of surviving tadpoles, measured total body length, and determined the proportion that survived.

To compare proportion of embryos and larvae surviving among habitat types, we used generalized linear models with a binomial distribution and log-link function incorporating habitat type, container location, and parental source as factors. We compared least-squares means among habitat types to determine where survival differed. We compared larval body length among habitat types using a two-factor analysis of variance with habitat type and parental source as factors. Waller-Duncan multiple comparison tests were used to determine which means differed.

Within each habitat type in each study area, we established three 25-m^2^ plots, centered on the container arrays. We measured forest overstory canopy cover, tree diameter at 1.1 m above the ground for trees ≥5 cm, and heights of three dominant trees, and we compared these characteristics among habitat types using *t*-tests.

### Treefrog predation

We studied treefrog predation from August–September 2011 (start of wet season) in Thailand, where *P. leucomystax* breeds from February to September (Heyer, 1973; Sheridan, 2008). We studied *P. megacephalus* from April–June 2011 (wet season) in Hong Kong, where the species breeds from March–August (Lau, 1998). In Hong Kong and in Thailand, we established arrays of 24, 12-liter containers, grouped as three sets of eight, in each habitat type and each associated with a set of *Microhyla* spp. containers. Thus, we had three replicates within each habitat type (intact forest, degraded forest, agriculture) within each study area (Hong Kong, Thailand). We randomly selected four of eight containers in each set and covered their tops with nylon mesh (1 mm^2^ openings) to prevent access to egg masses by flies. We used the same mesh to cover approximately 75% of the tops of the other four containers, permitting access to egg masses by flies. Containers were filled with 8 liters of filtered pond water. We collected egg masses of *Polypedates* spp. from nearby ponds and placed each egg mass on a small clump of grass attached to the side of the container, suspending the egg mass just above the water surface. For containers with partially-covered tops, egg masses were placed beneath the covered section for comparable shading.

We checked for hatching daily and when most eggs had hatched or when fly larvae had infested an egg mass, we counted numbers of surviving frog embryos and larvae, dead embryos, and fly larvae. We determined clutch sizes for egg masses not infested by flies. Because we could not determine clutch sizes for infested egg masses, we calculated survival for infested clutches as the number of surviving tadpoles from an infested clutch divided by the average clutch size of uninfested egg masses in each habitat type. Ten fly larvae from each infested egg mass were reared to adulthood for identification. To compare proportion of embryos surviving in each habitat type, we used generalized linear models with a binomial distribution and log-link function incorporating habitat type, location of containers, and parental source as factors. We compared least-squares means among habitat types to determine where survival differed. We used *t*-tests to compare numbers of fly larvae in accessible egg masses among habitat types. Within each habitat type, we measured forest characteristics as described above for *Microhyla* spp.

A scientific research permit (No. 0002/5011) was provided by the National Research Council of Thailand. This research was approved by University of Hong Kong Committee on Use of Live Animals in Teaching and Research (Permit #1830-09).

## Results

### Toad body condition and sex ratio

**Figure 1:**
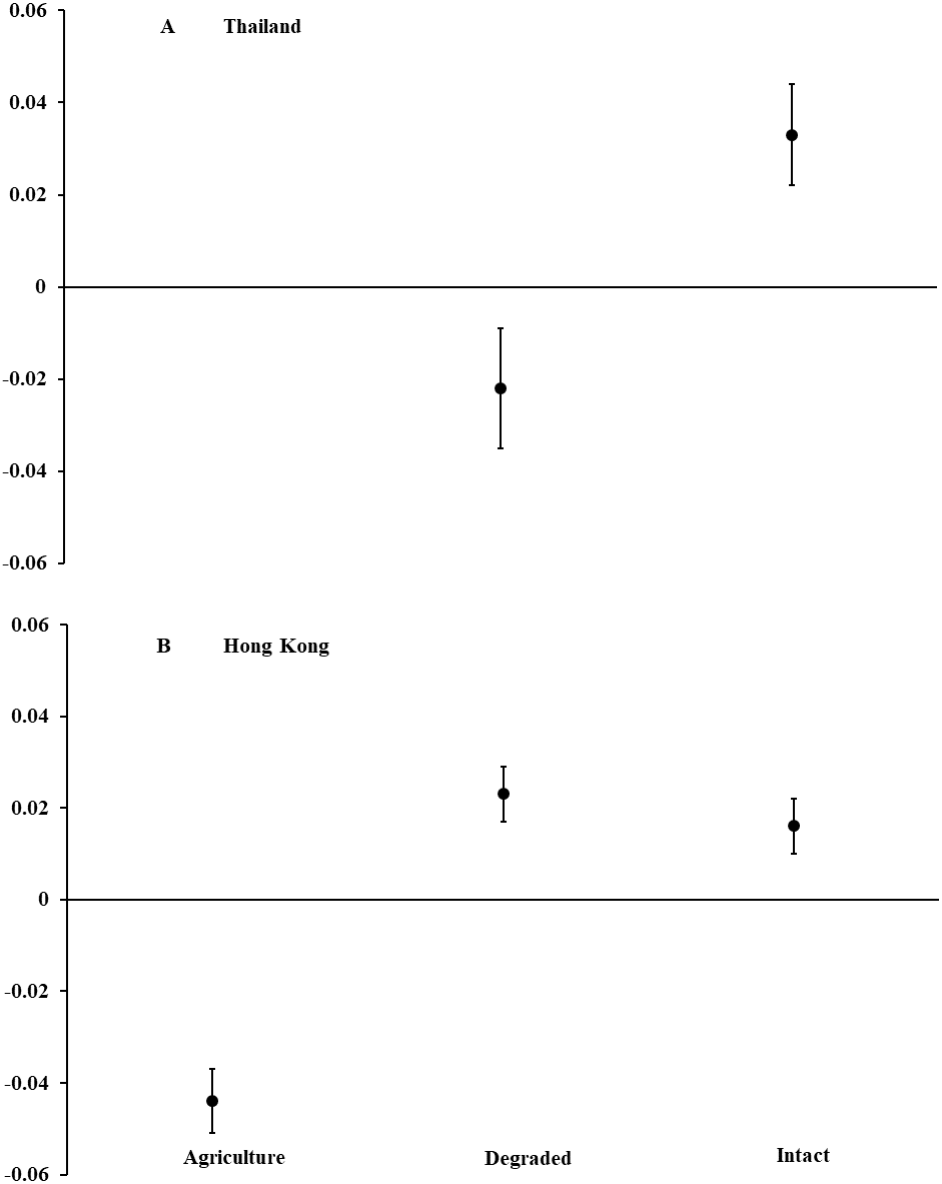
Body condition of Asian common toads (*Duttaphrynus melanostictus*). Toads were measured in agricultural land, degraded forest, and intact forest in (A) Thailand and (B) Hong Kong, and body condition was calculated as a relationship between body mass and length. Positive values indicate good body condition and negative values indicate poor body condition. We captured too few individuals (*n* = 4) in agricultural land in Thailand to include in analyses. Error bars indicate standard error.

In Thailand, we captured 120 *D. melanostictus* in intact forest (*n* = 47), degraded forest (*n* = 69), and agricultural land (*n* = 4). In intact forest, we documented a female-skewed sex ratio of 11 males and 31 females (M:*F* = 0.35; *χ*^2^ = 9.52, *P* = 0.002). In degraded forest, sex ratio was even with 26 males and 31 females (M:*F* = 0.84; *χ*^2^ = 0.44, *P* = 0.508). Only four toads were captured in agricultural land and so were not included in analyses. Body condition of all toads combined was higher (*F*_1,115_ = 9.26, *P* = 0.003; Fig. 1) in intact than in degraded forests but did not differ between males and females across habitat types (*F*_2,115_ = 0.26, *P* = 0.773). For both sexes combined, toads in intact forest averaged nearly 15% longer and 40% heavier than those in degraded forest. For all habitat types, female toads were a mean (±SE) length of 95.1 mm (±3.2, range = 54–194) and mass of 99.5 g (±7.0, range = 19–225). Males averaged 84.4 mm (±1.8, range = 58–119) and 74.8 g (±5.5, range = 23–203).

Vegetative structure differed among intact forest, degraded forest, and agricultural land. Canopy cover was 16–29% higher (*t*_1,29_ = 8.30, *P* < 0.001), tree diameters were 3–8 cm larger (*t*_1,29_ = 7.37, *P* < 0.001), and trees were 18–19 m taller (*t*_1,29_ = 15.71, *P* < 0.001) among plots in intact than in degraded forests. No trees were present in agricultural plots.

In Hong Kong, we captured 499 *D. melanostictus* in agricultural land (*n* = 156), degraded forest (*n* = 178), and intact forest (*n* = 165). Sex ratios were female-skewed in intact forest with 47 males and 77 females (M:*F* = 0.61; *χ*^2^ = 7.26, *P* = 0.007), and in degraded forest with 34 males and 92 females (M:*F* = 0.37; *χ*^2^ = 26.70, *P* < 0.001). In agricultural land, sex ratio was even with 25 males and 25 females (M:F 1.00). Body condition was higher (*F*_2,498_ = 33.40, *P* < 0.001; Fig. 1) in intact and degraded forests than in agricultural land. Body condition was similar between males and females across habitat types (*F*_1,301_ = 0.41, *P* = 0.523). Males and females combined averaged about 20% longer and 60% heavier in intact and degraded forests than in agricultural land. Across all habitat types, females averaged (±SE) 74.2 mm (±1.5, range = 50–103) in length and 59.8 g (±2.8, range = 8.0–170.6) in mass. Males averaged 60.3 mm (±0.8, range = 50–85) and 24.5 g (±1.1, range = 6.8–62.0).

Vegetation characteristics differed among the three habitat types. Canopy cover was 18–26% higher (*t*_1,29_ = 10.60, *P* < 0.001), and trees were 5–8 m taller (*t*_1,29_ = 7.23, *P* < 0.001) in intact than degraded forest. Tree diameters did not differ between intact and degraded forest (*t*_1,29_ = 1.89, *P* = 0.070). No trees occurred in agricultural land plots.

### Growth and survival of leaf litter frogs

**Figure 2:**
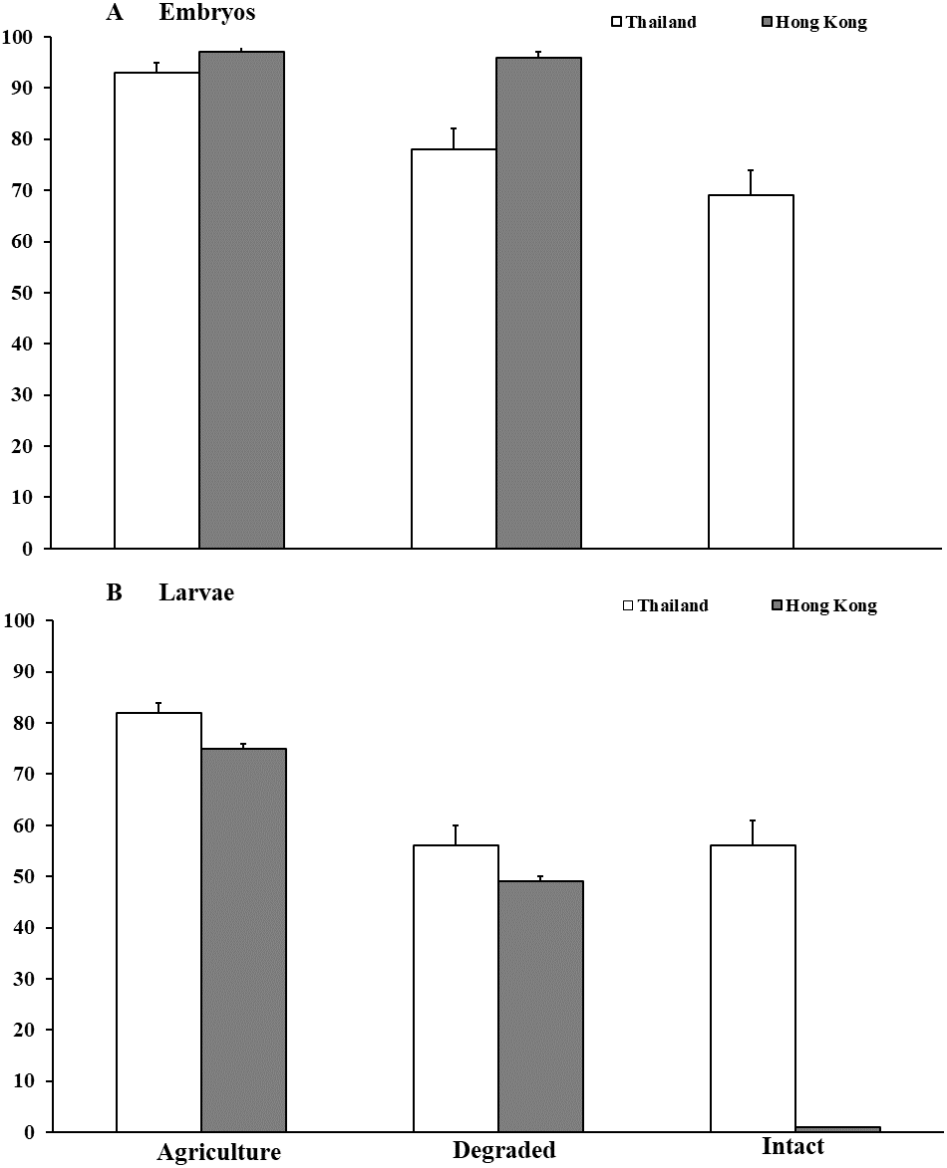
Embryonic and larval survival of *Microhyla heymonsi* and *M. fissipes*. (A) Survival of embryos to hatching and (B) survival of larvae to just prior to metamorphosis assessed in experiments conducted in agricultural land, and degraded and intact forests in Thailand and Hong Kong. Error bars indicate standard error.

In Thailand, survival of *M. heymonsi* embryos was 14% higher (*F*_2,49_ = 10.17, *P* < 0.001; Fig. 2) in agricultural land than in degraded forest (*z* = 2.39, *p* = 0.017) and 23% higher than in intact forest (*z* = 4.00, *P* < 0.001). Container location within the habitat type (*F*_1,49_ = 0.07, *P* = 0.791) and parental source (*F*_1,49_ = 0.03, *P* = 0.869) were not influential. Larval survival in agricultural land (*F*_2,48_ = 10.78, *P* < 0.001; Fig. 2) was 26% higher than in degraded forest (*z* = 3.91, *P* < 0.001) and 27% higher than in intact forest (*z* = 3.91, *P* < 0.001). Container location (*F*_1,48_ = 8.04 *P* = 0.0067) influenced survival, but parental source did not (*F*_1,48_ = 0.10, *P* = 0.751). Tadpoles were smaller (mean ±SE; *F*_2,43_ = 7.14, *P* = 0.002; Waller Duncan *P* < 0.05) in degraded forest (10.4 ± 2.0 mm) than in intact forest (12.7 ± 3.1 mm) and agricultural land (13.2 ± 2.3 mm). Larval growth was influenced by container location (*F*_2,43_ = 3.51, *P* = 0.039) but not by parental source (*F*_5,43_ = 0.52, *P* = 0.762).

Vegetative structure surrounding arrays in Thailand differed among habitat types. Canopy cover was 6–23% higher (*t*_1,5_ = 3.10, *P* = 0.030) and trees were 16–22 m taller (*t*_1,5_ = 7.42, *P* = 0.002) in intact than in degraded forests. Tree diameters were similar (*t*_1,5_ = 1.11, *P* = 0.330). No trees occurred in agricultural plots.

In Hong Kong, no *M. fissipes* embryos survived in intact forest compared with >95% survival in degraded forest and agricultural land (*F*_2,44_ = 2,459.78, *P* < 0.001; Fig. 2). Survival was not affected by container location (*F*_2,44_ = 1.01, *P* = 0.371), but parental source was marginally influential (*F*_5,44_ = 2.40, *P* = 0.053). Because no embryos survived in intact forest, we collected six egg masses, reared them to hatching in small containers in the agricultural area, and added hatchlings from those clutches to the larger containers in intact forest. Survival of tadpoles was about 25% higher (*F*_2,44_ = 68.42, *P* < 0.001; Fig. 2) in agricultural land than in degraded forest (*z* = 3.54, *P* < 0.001), and survival was <1% in intact forest (*z* = 6.67, *P* < 0.001). Container location (*F*_2,44_ = 1.29, *P* = 0.285) and parental source (*F*_5,44_ = 0.34, *P* = 0.889) were not important. Tadpoles (mean ± SE) in intact forest (7.75 ± 0.7 mm) were >50% smaller (*F*_2,29_ = 28.32, *P* < 0.001; Waller Duncan *P* < 0.05) than tadpoles in degraded forest (17.60 ± 1.1 mm) and agricultural land (18.97 ± 0.8 mm). Tadpole body size was not influenced by container location (*F*_2,29_ = 0.03, *P* = 0.971) or parental source (*F*_5,29_ = 1.92, *P* = 0.121).

Characteristics of vegetation around arrays differed among habitat types. Canopy cover was 37–44% higher (*t*_1,5_ = 9.86, *P* < 0.001) and trees were 5–6 m taller (*t*_1,5_ = 4.02, *P* = 0.016) in intact than in degraded forests. However, tree diameters did not differ (*t*_1,5_ = 1.18, *P* = 0.300). No trees occurred in agricultural plots.

### Treefrog predation

**Figure 3:**
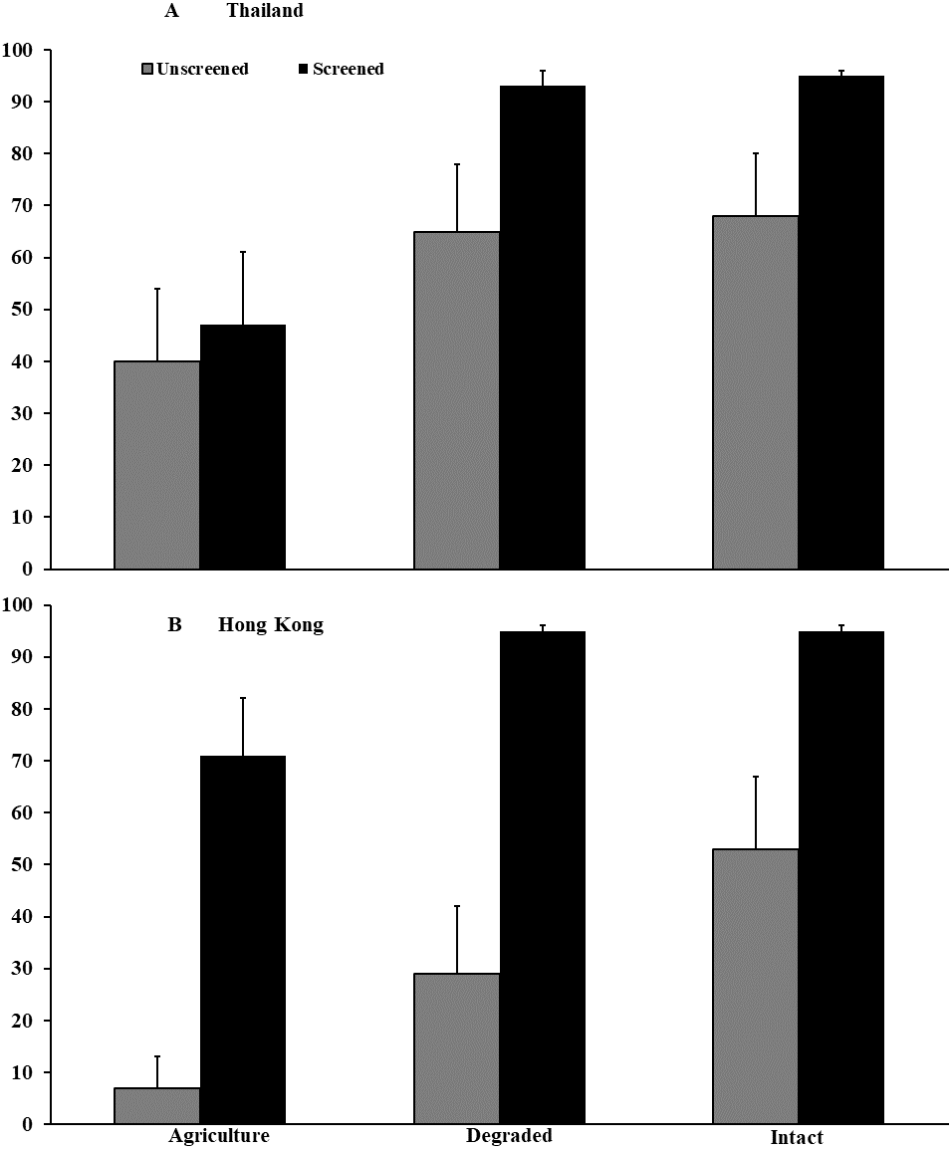
Embryonic survival of *Polypedates leucomystax* and *P. megacephalus*. Survival of embryos in egg masses that were accessible (unscreened) or inaccessible (screened) to predatory flies in the genus *Caiusa* quantified in experiments conducted in (A) Thailand and (B) Hong Kong. Error bars indicate standard error.

In Thailand, survival of *P. leucomystax* embryos to hatching, when accessible to flies, was 36–38% lower (*F*_2,46_ = 9.75, *P* < 0.001) in agricultural land than in intact (*z* = −3.24, *P* = 0.001) and degraded forests (*z* = −3.18, *P* = 0.0022). Survival was 20% lower (*F*_1,41_ = 13.34, *P* < 0.001; Fig. 3) in egg masses accessible to flies compared with those that were inaccessible, regardless of habitat type. We documented no interaction (*F*_2,46_ = 1.78, *P* = 0.180) between habitat type and accessibility by flies, and container location within habitat type did not influence survival (*F*_2,46_ = 1.60, *P* = 0.212). In agricultural land, fly larvae infested 56% of unscreened eggs masses, compared with 44% each in intact and degraded forests. Infested egg masses contained an average (±SE) of 36.0 (±21.8) fly larvae in agricultural land, 14.5 (±5.4) larvae in degraded forest, and 13.3 (±6.9) larvae in intact forest, but these numbers did not differ statistically (*t*_2,12_ = 0.85, *P* = 0.508). Egg masses inaccessible to flies averaged 559 eggs (±15.3, range = 383–729). Characteristics of vegetation differed among habitat types (see *Microhyla* spp. results).

In Hong Kong, survival of *P. megacephalus* embryos was 14–26% lower in agricultural land (*F*_2,43_ = 4.95, *P* = 0.012; Fig. 3) than in intact (*z* = −2.73, *P* = 0.006) and degraded forests (*z* =  − 2.09, *P* = 0.037), when accessible to flies. Survival in egg masses accessible to flies was 57% lower (*F*_1,43_ = 93.26, *P* < 0.001) than in those to which flies had no access, but there was no interaction between habitat type and accessibility by flies (*F*_2,43_ = 1.70, *P* = 0.195). Container location did not influence survival (*F*_2,43_ = 2.21 *P* = 0.123). Fly larvae infested 78% of accessible egg masses in agricultural land, 56% in degraded forest, and 44% in intact forest. Infested egg masses contained an average (±SE) of 13.9 (±3.6) fly larvae in agricultural land, 13.0 (±5.0) larvae in degraded forest, and 21.0 (±7.2) larvae in intact forest, and numbers did not differ among habitat types (*t*_2,15_ = 0.73, *P* = 0.594). Mean clutch size of egg masses inaccessible to flies was 331 (±16.2, range = 158–539). Vegetative structure surrounding the container arrays differed among habitat types (see *Microhyla* spp. results above). Flies were identified as *Caiusa coomani* in Hong Kong (Rognes, 2011) and as *C. coomani* and *C. violacea* in Thailand (Rognes, 2015).

## Discussion

We are in the midst of a biodiversity crisis, and that crisis extends to species once considered common and widespread, including among butterflies (Van Dyck et al., 2009) and moths (Conrad et al., 2006), birds (Elliott et al., 2010), bats (Gregory et al., 2007), marsupials (Frick et al., 2010), amphibians (Lehtinen & Skinner, 2006; Lindenmayer et al., 2011; Muths et al., 2003; White & Pyke, 1996), and other taxa. As humans’ activities continue to degrade remaining habitats, we must expand our research beyond documentation of occurrence and distributions. Several lowland amphibians in East and Southeast Asia, considered to be common and widespread, have been called ‘human commensals’, because although these species are thought to be abundant in intact, relatively well-protected forests, their populations persist in disturbed areas. We suggest that their occurrence in disturbed habitats does not indicate that their populations are unaffected by human-caused disturbance. We present evidence that in more disturbed habitats amphibians exhibited lower body condition, altered sex ratios, and experienced increased risk of predation. These results were comparable between two study areas located about 1,500 km apart.

We documented demographic parameters consistent with stable populations in intact forest. Toad populations exhibited higher mean body condition in intact forest. Reduced body condition can result from additional energy expenditures associated with increased physiological stress (Bosch, Martinez-Solano & Garcia-Paris, 2001; Homan et al., 2003), differences in longevity, or may be related to reduced feeding activity due to unsuitable microclimates or reduced prey availability or quality. Higher body condition in toads has been associated with higher quality habitats containing greater abundances of prey (Carey, 2005), which may be associated with moister conditions and more complex forest structure. Higher body condition also confers increased fitness, including higher fecundity (Sztatecsny & Schabetsberger, 2005) and higher adult survival (Reading & Clarke, 1995). We consistently documented female-skewed sex ratios in intact forest but an even sex ratio in some disturbed habitats. We believe that the sex ratios we documented are genuine and unlikely to be due to differences in habitat use by males and females outside of the breeding season. Sex ratios in populations of other frogs in protected, forested habitats have been shown to be female-skewed (Chan, Shoemaker & Karraker, 2014), and sex ratios of toads in disturbed habitats have been reported as male-skewed (e.g., Husté, Clobert & Miaud, 2006). Shifts in sex ratios reduce the effective population size and can lead to inbreeding and reductions in genetic diversity in populations over time (Frankham, 1995). Additionally, male-skewed sex ratios in disturbed habitats have been associated with declining populations (Grüebler et al., 2008; Hall, Henry & Bunck, 1999).

Ecological processes are often altered in disturbed habitats and such alterations can impact populations. We found that predator–prey relationships were modified in disturbed habitats, thereby impacting the demography of treefrogs. Specifically, we found that predation risk for amphibian embryos by flies was higher in disturbed habitats, resulting in lower survival to hatching. Habitat disturbance can lead to an increase in predators (Donovan et al., 1997), either by habitats becoming more accessible to predators or by cover for prey becoming scarcer, or a combination of the two. Increases in predator population size in disturbed habitats may overwhelm prey populations. In some cases, natural indicators of predation risk may be obfuscated in disturbed habitats, thereby increasing predation pressure (Shapira, Sultan & Shanas, 2008). Our findings of both demographic impacts and disruption of ecological processes in disturbed habitats may have implications for future population trends, in light of continued degradation of habitats by livestock grazing, fires (Langner & Siegert, 2009), woodcutting (Ross, 2001), and conversion to agriculture in the region (Achard et al., 2002). More critically, conversion of forests to oil palm plantations has exceeded 50% of some countries’ total land area in the past two decades (Koh & Wilcove, 2008).

Creation of canopy gaps by humans’ activities may benefit populations of some species. We found that survival in *Microhyla* spp. was highest in agricultural land in Thailand and Hong Kong. In peninsular Malaysia, *M. heymonsi* is more strongly associated with oil palm plantations, or open habitats, than mature secondary forests (Faruk et al., 2013). In southern China, *M. fissipes* is more abundant at disturbed sites than in intact rainforest (Behm, Yang & Chen, 2013). Many frogs in this genus are considered habitat generalists and prior to major degradation of forests in the region, these species probably bred only in forest gaps—open areas in forests caused by windfall of trees and subsequent pooling of water. Abundances of some species may have increased with expansion in the availability of open canopy breeding sites.

## Conclusions

For three of five species of amphibians, we documented negative signals of habitat loss and disturbance in their populations. The notion that populations of common and widespread species are healthy in disturbed habitats may more accurately reflect our perceptions of their populations in a way akin to a ‘shifting baselines syndrome’ (Pauly, 1995). Population trend information is not available for many species in the tropics and biologists continue to note their occurrence in disturbed habitats but many fail to look beyond occurrence. Although the species we examined persist in disturbed habitats, loss of forest cover may be exerting a chronic demographic toll on their populations. Numerous studies have demonstrated that forest degradation negatively impacts biodiversity (Sodhi et al., 2010) and calls have been made for preserving the remaining large patches of forest in the region. We urge conservation biologists to further explore the complex impacts of forest disturbance on animal populations, and to consider that populations of species appearing to be common, widespread, and tolerant of human disturbances may, in fact, be eroding over time.

##  Supplemental Information

10.7717/peerj.4220/supp-1 Data S1 Amphibian data from Hong Kong Raw data from Hong Kong on body condition of *Duttaphrynus melanostictus*, larval growth and survival of *Microhyla fissipes*, and embryonic survival of *Polypedates melanostictus.*

10.7717/peerj.4220/supp-2 Data S2 Amphibian data from Thailand Raw data from Thailand on body condition of *Duttaphrynus melanostictus*, larval growth and survival of *Microhyla heymonsi*, and embryonic survival of *Polypedates leucomystax.*

## Additional Information and Declarations

### Field Study Permissions

The following information was supplied relating to field study approvals (i.e., approving body and any reference numbers): A scientific research permit (No. 0002/5011) was provided by the National Research Council of Thailand.

### Data Availability

The following information was supplied regarding data availability: The raw data has been provided as Supplemental Files.
